# Comparison of hemodynamic changes and fetal outcome between normotensive and preeclamptic parturient undergoing elective cesarean section under spinal anesthesia: A prospective observational cohort study

**DOI:** 10.1016/j.amsu.2022.103829

**Published:** 2022-05-24

**Authors:** Sintayehu Mulugeta Tamiru, Abere Tilahun Bantie

**Affiliations:** a Department of Anesthesia, College of Health Science, Mekelle University, Mekelle, Ethiopia; b Department of Anesthesia, College of Medicine and Health Science, Addigrat University, Addigrat, Ethiopia

**Keywords:** Sever preeclampsia, Normal pregnancy, Spinal anesthesia, Neonatal outcome, Hemodynamic change

## Abstract

**Background:**

Maternal hypotension is a common problem during spinal anesthesia resulting in adverse maternal and fetal outcomes. According to theoretical knowledge, it is more common in severe preeclamptic parturients undergoing cesarean section with spinal anesthesia.

**Objective:**

To compare fetomother outcome in normotensive and severe preeclamptic parturients undergoing elective cesarean section under spinal anesthesia.

**Methodology:**

A prospective cohort study was conducted from Novembers to May 30, 2019 on 84 ASA II and III pregnant mothers. After preloading with 500 ml–1000ml crystalloids, a 0.5%isobaric bupivacaine of 10 mg–12.5 mg was administered for spinal anesthesia.

Vital signs (SBP, DBP, MAP and HR) were recorded every 3 min till 30 min, every 5 min then after. Neonatal Apgar scores at one and 5 min after birth and intraoperative fluids consumption were recorded. Data distribution was checked by Shapiro walk's test. Chi-square test was used to calculate the incidence of hypotension between groups; both paired and unpaired t-tests were also used to calculate the percent fall in blood pressure and heart rate from baselines of each group and intergroup respectively, and P-value less than 0.05 were considered statistical significance.

**Results:**

The incidence of hypotension (over a period of 30 min after spinal anesthesia) in the preeclamptic patients (31%) was less than that of the healthy parturients (59.5%). There was no statistically significant difference in heart rate of both groups before and after induction of spinal anesthesia. The 5th minute Apgar score recordings were also comparable between the groups.

**Conclusion:**

This study showed that the incidence and magnitude of spinal anesthesia-induced hypotension was less in severely preeclamptic parturient than healthy parturient who underwent elective cesarean delivery under spinal anesthesia and fetal outcome was comparable.

## Highlights

• Peak sensory block level was similar in both groups and no patients complained intraoperative pain and analgesics were not required supplemental.
• Base line SBP, DBP and MAP were significantly higher in severe preeclamptic group (p = 0.01).
• Mean gestational age of the fetus was significantly low in severe preeclamptic group (p = 0.01).
• Apgar score at 1st min(7–8 vs,8–9),volume of intra operative fluid use were statistically significant with p < 0.05

## 1 Introduction

### 1.1 Background

Pregnancy induced hypertension constitutes major cause of morbidity and mortality in developed and developing countries, and it is 5–10% of all pregnancies [1].Reported incidence of preeclampsia in obstetrics practice is 5–7% worldwide which is the third leading cause of maternal morbidity and responsible for 15–20% of maternal deaths worldwide [2], also the leading reason of maternal ICU admissions and principal cause of fetal morbidity and mortality [3,4] and in Ethiopia it holds 5.47%, which accounts for 19% of maternal deaths [5].

The exact cause is not known yet, but endothelial cell dysfunction has been implicated in its pathogenesis [6]. An increased plasma volume and a decrease in systemic vascular resistance are some of common physiologic changes during normal pregnancy [6], while an increase in systemic vascular resistance, lower plasma volume, hypertension and proteinuria are common manifestations of pregnancy with preeclampsia and it definitive management is termination of pregnancy [7].

Women with preeclampsia have an increased rate of cesarean section accompanied with high incidence of intrauterine growth restriction, fetal distress, and prematurity [8].

The available choices of anesthesia are general anesthesia or regional blocks, but each has its own unwanted effects on the maternal hemodynamics and consequently on the fetus.

General anesthesia is associated with risks like airway edema, difficulty with the airway or failed intubation, hypertensive response to direct laryngoscopy, and aspiration pneumonitis [[9], [10], [11]] [[9], [10], [11]] [[9], [10], [11]].

Spinal anesthesia-associated hypotension may occur in up to 64%–100% of pregnant women undergoing cesarean delivery which may produces nausea, vomiting, and light-headedness and It may also c decrease in utero placental blood flow and result in fetal acidosis [12,13].

Current clinical experience demonstrated relative safety of regional technique over general anesthesia for cesarean delivery in cases of severe preeclampsia according to recent studies [4,11,14] [[4], [11], [14], [15], [16], [17]] [[4], [11], [14], [15], [16], [17]].

There were inconsistent findings in incidence of hypotension, magnitude of hemodynamic changes, and on neonatal outcome (1st minute Apgar scores) between severely preeclamptic and healthy parturients [8,18,19]. While some studies conclude that there is no any differences in incidence of hypotension, magnitude of hemodynamic changes and neonatal outcome between severely preeclamptic and healthy parturients [12,20].

Although controversial, preloading or co-loading of colloid and crystalloid and administration of vasopressor agents are effective on reducing and treating spinal anesthesia induced hypotension in normotensive parturient [9,21]. But this interventions could increase risk of hypertension and iatrogenic pulmonary edema in preeclamptic patients [10,22,23].

Therefore, the present study was carried out in an effort to compare fetal out come and hemodynamic changes in patients with severe preeclampsia and healthy parturients undergoing spinal anesthesia for elective cesarean section.

## 2 Methods and materials

### 2.1 Study design, area and period

Prospective observational cohort study was conducted on ASA II and ASA III parturients at Ayder Comprehensive specialized Hospital, from Feb 2019–June 2019. Ayder is a teaching center for medicine and other health sciences. It has been giving referral and none-referral service for more than 8 million people including patients from Afar, and Northeastern parts of the Amhara Regional States. It provides a broad range of medical services to both in- and outpatients of all age groups. Ayder has a total capacity of about 500 inpatient beds in four major departments (internal medicine, surgery, pediatrics and obstetrics and gynecology). It has seven operating rooms and an average of 10 surgeries done per day. This study has been registered with a Research Registry UIN of researchregistry7766 at www.researchregistry.com) and reported according to STROCSS [24].

### 2.2 Source population

All mothers who underwent caesarean section delivery under spinal anesthesia during the study period.

### 2.3 Study population

All parturient who fulfilled the inclusion criteria that underwent caesarean section delivery under spinal anesthesia within study period.

### 2.4 Study variables

#### 2.4.1 Dependent variables (outcome variable)

Apgar score (1st and 5th minute), Blood Pressure, Heart rate and Vasopressor use were dependent variable while.

#### 2.4.2 Independent variable

Age, weight, height,ASA status,preoperative surgical diagnosis,duration of surgery,base line heart rate, base line blood pressure,total amount of fluid, blood loss,utero-tonic agent,gestational age, parti and gravidity were independent variables.

### 2.5 Inclusion and exclusion criteria

#### 2.5.1 Inclusion criteria

Non-laboring, ASA (physical status II, III)**,** singleton pregnancy**,** elective cesarean section were considered as inclusion criteria.

#### 2.5.2 Exclusion criteria

Patients with chronic hypertension, cardiac, renal or endocrine disease, pregnancy with congenital anomaly, allergy to local anesthetics, abruption placentae or placenta previa were excluded from the study.

### 2.6 Sample size determination

Two independent sample size formula was used from mean difference of outcome variables (SBP, DBP, MAP, HR, Apgar score and the incidence of hypotension). The larger result was used from (mean difference of DBP). The lowest decrease of DBP in healthy groups was 29.4 ± 15.3% and lowest decrease of DBP in severe pre-eclamptic groups was 21.01 ± 11.5% [25].

The sample size was calculated by program software G*power version 3.1.9.2 (Power = 80, α = 0.05,β = 0.20).Final sample size was 42 mothers for each group.

### 2.7 Sampling technique

All mothers delivered by cesarean section under spinal anesthesia during the study period and fulfilled inclusion criteria were selected consecutively until the required number has been achieved.

### 2.8 Data quality control

To assure the quality of the data, a pretest was done on 5% of the sample after that the questionnaires were modified accordingly and providing training and orientation about the objectives and relevance of the study, each items included in the study tools and the whole process of data collection for two BSc anesthetists as principal data collectors, one nurse as assistant and supervisors data was collected using pretested questionnaires.

Supervisors checked each questionnaire daily with further cross check by principal investigator for completeness and consistency of data.

### 2.9 Data processing and analysis

The data was analyzed with Statistical Package for the Social Sciences (SPSS) version 20 computer program (IBM) after it was cleaned and coded. Data was summarized in mean and standard deviation and results were display by using texts, tables and figures.

Chi-square test was used to calculate the incidence of hypotension between groups; both paired and unpaired t-tests were used to calculate the mean differences of both blood pressure and heart rate from their corresponding baselines of each group and intergroup respectively, data distribution was checking using Shapiro walk's test. P value less than 0.05 (p < 0.05) was considered as significant.

### 2.10 Anesthesia management standard protocols

As standard of care 10 mg of IV metoclopramide was given for all parturients at the morning of the surgery. In addition all severe preeclamptic mothers had taken magnesium sulphate 4 gm for seizer prophylaxis and hydralazine of 20 mg (daily dose).

At arrival patients in the operating room, patient charts were checked and reviewed (antihypertensive agents were recorded), a brief clinical examination was done and standard ASA monitors were attached including electro cardio gam, pulse oximetry and, none invasive blood pressure monitoring.

Parturients were preloaded with normal saline or lactate Ringer's solution, of at least 500 ml over 15–20 min before the anesthesia, with patient in left lateral position. Baseline SBP, DBP, MAP and HR were calculated as mean of two consecutive measurements 2 min apart. After cleaning the site aseptically with iodine and alcohol and skin infiltration with 2 ml of 2% lidocaine, spinal anesthesia was administered with, 0.5% (2.5 ml or 12.5 mg) of isobaric bupivacaine by a 24 or 25 gauge spinal needles (Quincke) in L3-4 or L4-5 vertebral interspaces through a midline approach in sting position by confirming free flow of cerebrospinal fluid, and the patient returned to supine position with left uterine displacement and slight (10-15^0^) head up position. Surgery was started after adequate sensory block has been confirmed. Immediately after delivery, oxytocin 20 IU was started as infusion and fetus wellbeing was assessed using APGAR scores at 1^st^and 5^th^minutes.

Since arrival at operation room, Patients were followed for 24 h to asses fetal and maternal out coms like vital sign change like SBP, DBP, MAP and HR for mother AGAR score for neonate.

## 3 Results

### 3.1 Socio demographic and baseline vital sign

Eighty four patients (severe preeclampsia = 42 and healthy = 42) were involved in analysis. Peak sensory block level was similar in both groups and no patients complained intraoperative pain and analgesics were not required supplemental.

Demographic data like age, weight, and total preoperative administered fluid and baseline heart rate were comparable between the group p > 0.05. But base line SBP, DBP and MAP were significantly higher in severe preeclamptic group (p = 0.01) and mean gestational age of the fetus was also significantly low in this group (p = 0.01) (Table 1).

**Table 1:** Demographic and baseline vital sign between the groups at Ayder comprehensive specialized hospital Nov1 to May 2019.

Variable	Normotensive (n = 42)	Preeclamptic (n = 42)	P-value
Age, year(mean ± SD)	29.3 ± 4	30.8 ± 3.8	0.09
Weight, kilograms(mean ± SD)	68.8 ± 8.6	72.2 ± 8.1	0.06
Height, meters(mean ± SD)	1.64 ± 0.6	1.64 ± 0.5	0.94
Gestational age, week(mean ± SD)	39 ± 1.07	35.5 ± 1	0.01**
Baseline SBP, mmHg(mean ± SD)	125.8 ± 11.8	161.3 ± 10.1	0.001**
Baseline DBP, mmHg(mean ± SD)	75.8 ± 11.4	104.6 ± 9.5	0.001**
Baseline MAP, mmHg(mean ± SD)	91.3 ± 10	123.5 ± 7.9	0.001**
Baseline HR, b/min(mean ± SD)	92 ± 10.6	91.8 ± 10.5	0.95
IV fluid preloaded, ml(mean ± SD)	481.7 ± 177	446.4 ± 164	0.348

** = statistically significant, mean ± SD = mean and standard deviation (unpaired T-test was used for analysis).

### 3.2 Intraoperative variables between groups

The sensory blocked levels, volumes of estimated blood loss, Apgar score at 5^th^min and duration of surgery were comparable between the two groups, while Apgar score at 1st min(7–8 vs,8–9),volume of intra operative fluid use (1353.6 ± 284 ml vs 1716.7 ± 431 ml) in severe preeclamptic and normal pregnancy group respectively were statistically significant with p < 0.05 (Table 2).

**Table 2:** Intraoperative variables between the groups at Ayder comprehensive specialized hospital Nov1 to May 2019.

Variable	Normotensive(n = 42)	S.preeclamptic(n = 42)	P-value
Upper level of sensory block(*)	T4(T4-T6)	T4(T4-T6)	0.601
APGAR score, 1^st^min(*)	8(8–9)	8(7–8)	0.004**
APGAR score, 5^th^min. (*)	9(9–10)	9(9–10)	0.737
Intraoperative crystalloid, ml	1716.7 ± 431	1353.6 ± 284	0.001**
Estimated blood loss, ml	443.6 ± 80	473.8 ± 74	0.078
Duration of surgery, min.	30.1 ± 3	29.8 ± 2	0.588

* Mann Whitney u-test →median (interquartile range), ** = statistically significant, mean ± standard deviation (unpaired T-test) →Values were expressed as median (interquartile range) for the Mann Whitney u-test and mean ± standard deviation for unpaired T-test.

### 3.3 Mean% changes of mean arterial pressure and heart rate after spinal anesthesia between the groups

The magnitude of changes (mean) in MAP in the first 9 min after spinal anesthesia and the incidence of hypotension were higher with statistical significant (p < 0.05) in normotensive group (59.5%) than the severely preeclamptic group (31%).The magnitude of changes(mean) in heart rate of both groups were not statistically significant throughout all time (p > 0.05).

### 3.4 Mean% changes of mean arterial pressure, systolic blood pressure and diastolic blood pressure after spinal anesthesia

**Fig. 1:**
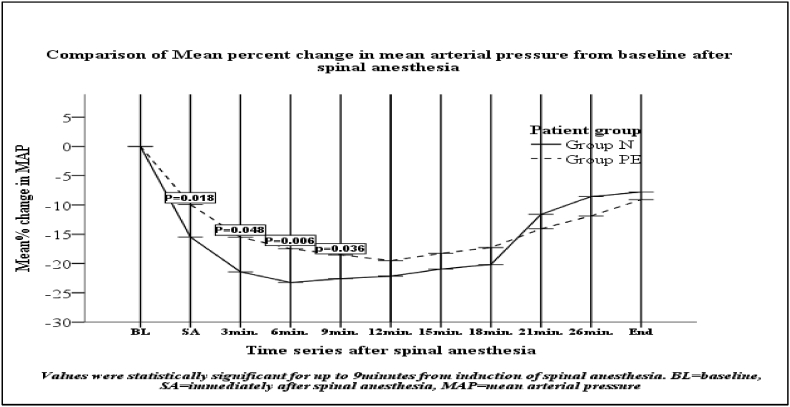
Mean% fall of mean arterial pressure after spinal anesthesia between the groups at Ayder comprehensive specialized hospital Nov1 to May 2019.

As indicated in Fig. 1, Fig. 3 the percent falling in MAP and diastolic blood pressure immediately after spinal anesthesia at 3min, 6 min and 9 min were higher in normotensive group with statistical significant (p < 0.05) than severe preeclampsia group.

**Fig. 2:**
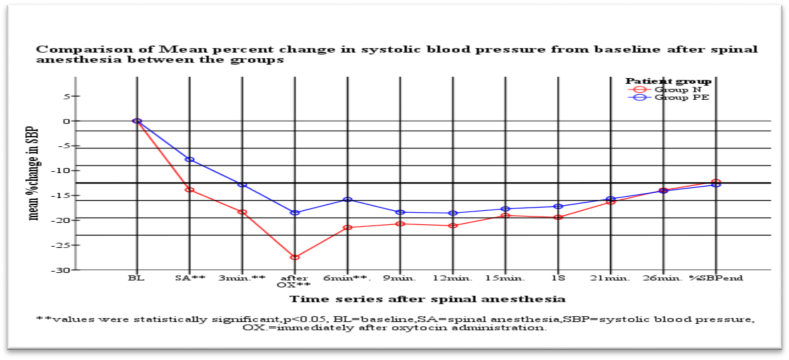
Comparison of percent fall in systolic blood pressure from baseline after spinal anesthesia between the groups at Ayder comprehensive specialized hospital Nov1 to May 2019.

**Fig. 3:**
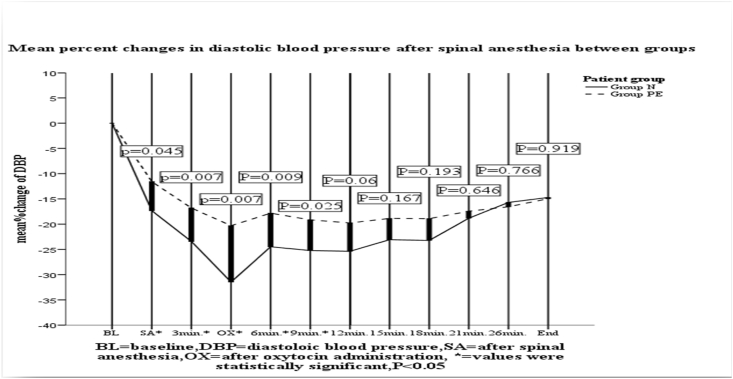
Comparison of mean %change in diastolic blood pressure from baseline after spinal anesthesia between the groups at Ayder comprehensive specialized hospital Nov1 to May 2019.

There were no statistically significant change in derangement in systolic blood pressure in all time and 9 min after spinal anesthesia till the end of surgery in mean (MAP) and diastolic blood pressure between the groups (Fig. 1, Fig. 2, Fig. 3).

## 4 Discussion

The reported incidence of spinal anesthesia-induced maternal hypotension is varied due to inconsistent definition among several studies, claimed to be between 7% and 89.2% [15,26].

Our study revealed that, in the first 9 min after spinal anesthesia; SBP, DBP and MAP decreased in both groups, but more in the normotensive groups as compared to the severely preeclamptic one. But there was no statistically significant difference in heart rate between groups. The incidence of hypotension after spinal anesthesia in preeclamptic patients (31%) was less than that of the healthy parturients (59.5%), despite the former receiving smaller volumes of intravenous fluids **(**Table 1**)**, (1353.6 ± 284 ml versus 1716.7 ± 431 ml) (P = 0.001).The mean one-minute Apgar scores in the patients with severe preeclampsia were significantly lower than those of the healthy parturients (8(7–8) vs 8(8–9)) with p = 0.004, but the 5th minute Apgar score recordings were comparable between the groups (p = 0.737).

A prospective study by Sivevski et al.in Republic of Macedonia found that the blood pressure(SBP,DBP and MAP) falls (%) from baseline were significantly greater in the healthy parturients compared to those with severe preeclampsia and the incidence of hypotension in the preeclamptics was 25% compared to 53% in healthy parturients [15].The result of this study was consistent with our finding.

Our finding also in line with a prospective comparative study done by Aya et al. on sixty parturient, which showed that the incidence of hypotension in the severely preeclamptic parturient was16.6% and 53.3% in normotensive groups, in spite of receiving less crystalloid fluids and higher dose of 0.5%bupivacaine.

Another study by Chowd hury et al. found that minimum SBP, DBP and MAP recorded were always higher in the preeclamptic group compared to the normotensive group. The percentage fall in MAP calculated from baseline was also less in the preeclamptic group. This was comparable with our study.

Ishrat and Raja by their prospective study concluded that, preeclamptic patients experienced less hypotension than healthy parturients following spinal anesthesia and fall in DBP and MAP were significantly higher in healthy parturients [25]. Our study results were also similar to their study. Nikoosersht et al. in Iran, reported that the incidence of hypotension in severely preeclamptics was found to be significantly lower in comparison to the rate among healthy parturients (55% vs 89%),despite the normotensive received more volumes of intravenous fluids (2.5 versus 2.4 lit.). This study found high incidence of hypotension in both groups. They defined (hypotension ≥25% decline to baseline MAP vs. ≥ 30% in our study) might explain the reason why the incidence of hypotension was higher in their study.

According to a study by Karuna and Pallavion a difference between normotensive and preeclamptic groups was not found on the occurrence of hypotension, decrement of blood pressure, vasopressor use, or newborn well-being after spinal anesthesia using 0.5% hyperbaric bupivacaine 2 ml (10 mg).This was in contrary to our study, and the reason could be the small dose of the drug [12].

The mean values of HR did not change significantly in both groups throughout all time intervals after the induction of spinal anesthesia, but intraoperative values were slightly higher than their baseline values in both groups (the negative values in Table 3).This finding was in line with other studies [15,25,27].

**Table 3:** Mean% changes of mean arterial pressure and heart rate after spinal anesthesia between the groups at Ayder comprehensive specialized hospital Nov1 to May 2019.

Variables over time series		Normotensive(n = 42)→group N	Preeclamptic(n = 42) group PE	P-value
After preload	MAP (%)	0.2 ± 4	1 ± 4	0.109
	HR (%)	−4±8	−4±6	0.738
After spinal	MAP	16 ± 13	10 ± 6	0.018**
	HR	−3±13	−8±7	0.07
3 min	MAP	21 ± 12	15 ± 7	0.048**
	HR	5 ± 13	8 ± 9	0.180
6 min	MAP	23 ± 12	17 ± 5	0.006**
	HR	3 ± 13	2 ± 10	0.637
9 min	MAP	23 ± 10	18 ± 8	0.036**
	HR	5 ± 14	7 ± 10	0.56
12 min.	MAP	22 ± 13	19 ± 8	0.263
	HR	5 ± 11	7 ± 11	0.425
15 min.	MAP	21 ± 13	18 ± 7	0.259
	HR	6 ± 10	7 ± 11	0.600
18 min.	MAP	20 ± 14	17 ± 7	0.232
	HR	−4±10	−6±10	0.125
21 min.	MAP	12 ± 8	14 ± 6	0.128
	HR	−3±11	−5±9	0.45
26 min.	MAP	9 ± 7	11 ± 5	0.068
	HR	−5±10	−8±8	0.155
At the end of surgery	MAP	8 ± 8	9 ± 6	0.403
	HR	−4±11	−5±10	0.353
Incidence of hypotension		59.5%(25/42)	31%(13/42)	0.015*

** Statistically significant values, Unpaired *t*-test used for analysis; Parameters were expressed as Mean ± Standard deviation, MAP = mean arterial pressure, HR = heart rate.

In terms of vasopressor use, only two patients from the normotensive group were treated with 10mcg of adrenaline, and this was not adequate for comparison.

Several factors might have contributed to less incidence of hypotension in the severely preeclamptic patients. An obvious factor is the large difference in gestational age between the study groups. Indeed, healthy parturients carrying a larger fetus may be at increased risk of aorto caval compression (higher gestational age in the normotensive would correspond to large fetal weight).

In addition, by dilating epidural blood vessels, the aortocaval compression could facilitate the cephalic spread of local anesthetics, leading to a higher upper level of spinal blockade in healthy parturients. Although the upper sensory levels were similar in both groups, the aortocaval compression may, at least in part, account for the increased incidence and severity of hypotension in the healthy parturients in our study.

Another explanation could be partly due to damaged vascular endothelium, as seen in severe PE, which produces increased amount of endogenous vasopressor like thromboxane and endothelia, resulting in persistent vasoconstriction. This phenomena is not altered even after SAB, resulting in less hemodynamic alterations; which contrasts with normal pregnancy, where altered balance of vascular tone, reduced response to endogenous vasopressors and increased synthesis of vasodilator prostaglandins and nitric oxide, make them very sensitive to spinal anesthesia, producing hypotension after spinal anesthesia [28].

In terms of fetal outcome, the mean 1-min Apgar scores in the patients with severe preeclampsia were significantly lower than those of the healthy parturients (8(7–8) vs 8(8–9) with p = 0.004, but the 5th minute Apgar score recordings were comparable between the groups (p = 0.737).

The cause of the lower mean 1-min Apgar scores in newborns from severely preeclamptic women may be that they had lower gestational age (35.5 ± 1 vs 39 ± 1.07,P˂ 0.001) and chronic utero placental insufficiency, which results in intrauterine growth restriction.

But studies found comparable 1st and 5th minute Apgar score results in both severely preeclamptic and normotensive groups. The explanation for this could be advanced antenatal care and use of vasopressor intraoperative [2,29].

Regarding fetal well-being, it had been theorized that the sympathectomy attributable to spinal anesthesia could significantly decrease utero placental blood flow in preeclamptics and lead to worse neonatal outcomes. Conversely, several studies supporting the safety of spinal anesthesia in these patients have been published, and neuraxial anesthesia for labor pain relief has even been shown to increase placental blood flow in patients with severe preeclampsia [20].

### 4.1 Implication for practice

Body of evidence reviled that better control of the perioperative hemodynamic changes can lead to reduction in perioperative morbidity and mortality in obstetric patients. Therefore, a mitigating strategy is required by different stake holders to control the hemodynamic changes and newborn well-being in patients with severe preeclampsia and healthy parturients undergoing spinal anesthesia for cesarean section.

### 4.2 The implication for further research

The present study reviled spinal anesthesia can provide reliable and fast anesthesia, especially in busy setups like in our institution. How there is a variability of anesthetists’ interest on choosing better anesthetic technique for severely preeclamptic patients i.e. some use general anesthesia by the fear that spinal anesthesia could cause severe hypotension and others abandon using general anesthesia for its complications. Therefore, further randomized controlled trials are required to validate the safety of spinal anesthesia for pre-eclamptic patients and provide a firm conclusion.

## 5 Limitation of the study

Anti-hypertensive agents were not protocoled and oxytocin drip was not strictly controlled and smaller sample size may affect our results.

## 6 Conclusion and recommendations

### 6.1 Conclusion

Our study showed that the incidence and magnitude of spinal anesthesia-induced hypotension was less in severely preeclamptic parturient than healthy parturient while fetal outcome was comparable.

### 6.2 Recommendations

Spinal anesthesia is safe for severely preeclamptic parturient.

We recommend further studies with the antihypertensive and uterotonic agents controlled (protocoled) on the study setting.

## Ethical approval

Ethical clearance and approval was obtained from the ethical review committee Mekelle University, Health Science College. Permission was obtained from medical director of Ayder comprehensive specialized hospital to conduct the research. The study was undertaken on the basis of the patients’ wish that in all circumstances by obtained informed oral consent. There was no coercion, and or no incentives to be involved in the study.

## Sources of funding

The study was self-funded.

## Author's contributions

Sintayehu Mulugeta and Abere Tilahun: Designed the study, supervised the data collection, performed the analysis, interpretation of data, drafted the manuscript and final approval of the revision for publication. Both authors also read and approved the final manuscript.

## Research registration unique identifying number (UIN)

Researchregistry 7766.

## Guarantor

Both authors.

## Provenance and peer review

Not commissioned, externally peer-reviewed.

No funding was obtained from any organization.

## Declaration of competing interest

No conflict of interest to declare.

## Abbreviations

ASA =: American Society of Anesthesiologists
Ug: Microgram
CS =: Caesarean Section
DBP =: Diastolic Blood Pressure
HR =: Heart rate, IV = Intravenous
MAP =: Mean Arterial Pressure
SA =: Spinal Anesthesia
SBP =: Systolic Blood Pressure
SVR =: Systemic Vascular Resistance
SPHMMC: Saint Paul's Hospital Millennium Medical College
ICU =: Intensive Care Unit, SAB = Sub-Arachnoid Block
APGAR: Appearance, Pulse, Grimace, Activity and Respiration of a baby
NIBP: Non-invasive blood pressure (measurement)
